# Femoral antetorsion after calcar‐guided short‐stem total hip arthroplasty: A cadaver study

**DOI:** 10.1002/jor.25228

**Published:** 2021-12-06

**Authors:** Josef Hochreiter, Gernot Böhm, Johann Fierlbeck, Conrad Anderl, Marco Birke, Peter Münger, Reinhold Ortmaier

**Affiliations:** ^1^ Department of Orthopedic Surgery, Ordensklinikum Linz GmbH Barmherzige Schwestern Hospital Linz Austria; ^2^ Department of Orthopedics and Traumatology Paracelsus Medical University Salzburg Austria; ^3^ Department of Diagnostic and Interventional Radiology Ordensklinikum Linz GmbH Linz Austria; ^4^ Institute for Clinical Innovation Paracelsus Medical University Salzburg Austria; ^5^ Institute of Anatomy and Cell Biology Paracelsus Medical University Salzburg Austria; ^6^ Mathys Ltd Bettlach Bettlach Switzerland; ^7^ Research Unit of Orthopedic Sports Medicine and Injury Prevention, Institute for Sports Medicine, Alpine Medicine and Health Tourism, UMIT Private University for Health Sciences Medical Informatics and Technology GmbH Hall in Tirol Austria

**Keywords:** cadaveric study, calcar‐guided short stems, femoral antetorsion, total hip arthroplasty

## Abstract

Calcar‐guided short stems in total hip arthroplasty (THA) permit surgeons to successfully reconstruct postoperative femoroacetabular offset, accurately restore leg length, and adequately re‐establish a wide range of caput‐collum‐diaphyseal angles. However, their effect on femoral antetorsion is less known. Indeed, controlling antetorsion of the femoral stem can be challenging because of the differences in individual femoral geometry and curvature. Therefore, we investigated if calcar‐guided short‐stem THA alters femoral antetorsion and compared it with conventional‐stem THA. Using 12 Thiel‐fixed, full‐body cadaver specimens from donors without known hip disorders, we compared an uncemented calcar‐guided femoral short‐stem prosthesis with an uncemented conventional straight‐stem prosthesis. In a paired study setup, each specimen received a calcar‐guided short stem on one side and a conventional stem on the other. On the acetabular side, all specimens received a press‐fit, monobloc acetabular cup. Femoral antetorsion angles were measured using the Waidelich method, and pre‐ and post‐operative angles of both sides were recorded. The mean preoperative femoral antetorsion angles were similar in both groups (24.8°  ± 7.5° vs. 23.8° ± 6.1°, *p* = 0.313). Mean postoperative femoral antetorsion angles were 23.0° ± 5.5° in short‐stem and 13.5° ± 7.1° in conventional‐stem hips. Short‐stem hips had a small but nonsignificant difference in femoral antetorsion angles pre‐ and post‐operatively (1.8° ± 3.2°, *p* = 0.109), while the difference for conventional‐stem hips was much larger and highly significant (10.3° ± 5.8°, *p* < 0.001). Calcar‐guided short‐stem THA effectively restores femoral antetorsion. However, how this affects long‐term clinical outcomes and complications warrants further exploration.

## 1 INTRODUCTION

The goal of total hip arthroplasty (THA) is to restore normal hip anatomy and biomechanics. To achieve this objective, crucial factors affecting the patient's anatomy, including leg length, caput‐collum‐diaphyseal (CCD) angle, femoroacetabular offset, and femoral antetorsion, must be considered.^1^, ^2^, ^3^, ^4^

To date, several hip prosthesis designs have been developed to accurately reconstruct the hip joint. Among them are calcar‐guided short stems, which were designed to optimally adapt to the proximal femur's anatomy and restore hip biomechanics. Their curved design renders individual stem positioning possible in a wide range of varus and valgus alignments,^5^ a conclusion confirmed by a clinical study in which calcar‐guided short‐stem prostheses allowed for accurate hip joint reconstruction in the studied patient population.^6^ In addition, calcar‐guided short stems permit surgeons to successfully reconstruct postoperative femoroacetabular offset, accurately restore leg length, and adequately re‐establish a wide range of CCD angles in most patients, contributing to the restoration of anatomical hip geometry and favorable midterm clinical outcomes.^1^, ^5^, ^6^, ^7^, ^8^, ^9^, ^10^, ^11^

In addition to the abovementioned parameters referring to the anteroposterior view, antetorsion of the femoral component also plays an important role in proper hip stability.^2^, ^4^, ^12^, ^13^ Both excessive antetorsion and retrotorsion can lead to impingement and instability.^13^ Correct component torsion is therefore necessary to achieve an impingement‐free range of motion and prevent well‐known complications of THA such as instability, dislocation, and component wear.^2^, ^13^, ^14^

Conventional stems have led to substantial reduction in femoral antetorsion angles after THA compared with preoperative values.^4^ However, to our knowledge, the long‐term clinical significance of this has not been studied. Although calcar‐guided short stems allow good anatomical reduction in the anteroposterior view, their effect on femoral antetorsion is less known.

For this reason, we carried out an in vitro study to examine (1) whether calcar‐guided short‐stem THA alters postoperative femoral antetorsion and (2) how calcar‐guided short‐stem THA compares with conventional‐stem THA. We hypothesized that the femoral antetorsion angle would not change significantly from preoperative values after calcar‐guided short‐stem THA.

## 2 METHODS

### 2.1 Selection and preparation of cadaver specimens

The study included 12 Thiel‐fixed full‐body cadaver specimens from donors without known hip disorders, such as dysplasia, fracture sequelae, and proximal femoral bone defects, used according to institutional guidelines. The cadavers were prepared according to the standard protocol described by Thiel,^15^ using modified Thiel solutions.^16^

### 2.2 Surgical approach and implants

An anterolateral approach was used in the supine position without a traction table in all cadaver specimens. In all procedures, the hip was externally rotated. With 90° of knee flexion, the ipsilateral lower leg thus ended up in the horizontal plane, parallel to the operation table.

Both conventional and short stems were implanted according to the accompanying surgical techniques. In conventional stems, the implants were inserted with an antetorsion perpendicular to the lower leg, and the femoral neck did not guide the implant during insertion. In short stems, the implants were inserted along the partially preserved femoral neck (so‐called “calcar‐guided” implant insertion), which determined the final position of the implant.

Three senior orthopedic surgeons (J.H., C.A., and R.O.) from the same clinic operated on four cadaver specimens each (left and right side). The three surgeons had operated on over 100 cases using both implants included in this study, and therefore, no learning curve was associated with calcar‐guided short‐stem implantation.^17^

On the femoral side, an uncemented calcar‐guided femoral short‐stem prosthesis (optimys stem; Mathys Ltd Bettlach) was implanted and compared with an uncemented conventional straight‐stem prosthesis similar to the Zweimüller design (CBH stem; Mathys Ltd Bettlach). Instead of actual implants, three‐dimensionally printed stems made of polylactide and 28‐mm trial heads made of polyphenylsulfone^18^ were used to minimize image artifacts. However, the implant bed was prepared using standard instruments. On the acetabular side, a press‐fit, monobloc acetabular cup (RM Pressfit; Mathys Ltd Bettlach) was implanted in all cases.

All cadaver specimens were randomly assigned to the surgeons (four specimens each). In a paired study setup, calcar‐guided short‐stem implants were implanted on one side and conventional‐stem implants on the other based on random side allocation, making sure that both groups had the same number of left and right implants.

### 2.3 Image acquisition and measurement of femoral antetorsion angles

All pre‐ and post‐operative measurements were recorded with a computed tomography (CT) scanner (Somatom Emotion 6, Siemens) using the corresponding three‐dimensional imaging software (Syngo Via, Siemens). All scans were performed helically with a vertical gantry using a layer thickness of 1.25 mm. We centered the specimens with feet parallel in a supine position and scanned them from the iliac crest to the knee joint. All images were reconstructed with an overlapping reconstruction technique to obtain images with a slice thickness of 0.8 mm. We used a hard kernel (UH90) and a bone window setting to obtain high‐quality images for radiographic evaluation.

**Figure 1:**
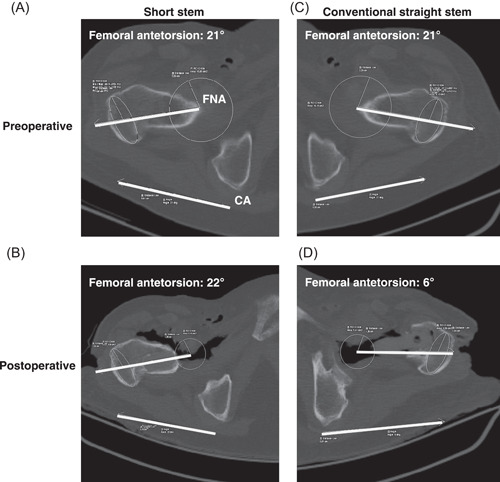
Pre‐ and post‐operative CT scans of one specimen showing femoral antetorsion angles (°) using the Waidelich method. A Short‐stem prosthesis was implanted in the right hip (A, C); a conventional straight‐stem prosthesis was implanted in the left hip (B, D). The circles represent the femoral head or the implanted prosthesis head, the ellipses the greater trochanter area where the posterior protrusion is clearly defined, and the bold straight lines the measured femoral neck and epicondylar axes. CA, condylar axis; FNA, femoral neck axis

Femoral antetorsion was measured with the Waidelich method, which uses superimposed CT transverse slices.^19^ Femoral antetorsion was calculated as defined previously by measuring the angle between two lines: The first line connected the center of the femoral head or the head of the implanted prosthesis to the center of an ellipse around the greater trochanter. The second line connected the posterior aspect of the medial and lateral femoral condyles.^14^, ^20^ To summarize briefly: we began by drawing a circle around the femoral head or the implanted prosthesis head, and then drew a straight line along the dorsal edge of the femoral condyles in the distal femoral area (Figure 1). Next, we drew an ellipse in the greater trochanter area where the posterior protrusion is clearly defined, transferred the head circle and condylar line to the height of the ellipse, and then drew a second straight line between the center of the ellipse and the center of the circle. Finally, we measured the angle between the lines, representing the femoral neck axis and the epicondylar axis, and documented the femoral anteversion angle.

### 2.4 Statistical analysis

We used descriptive statistics (means, *SD*s, and 95% confidence intervals) to describe specimen characteristics and outcome variables at the measurement point. Paired *t* tests were used to determine pre‐ and post‐operative differences in each specimen, and the Shapiro–Wilk test was applied to test for normality. The sample size was determined based on a paired scenario assuming a within‐patient correlation of 0.85, yielding power of 80%. Statistical analysis was performed with SAS version 9.4 (SAS Institute Inc). A value of *p* < 0.05 (two‐sided) was considered statistically significant.

## 3 RESULTS

Baseline characteristics of the donor population are described in Table 1. The mean preoperative femoral antetorsion angles were similar in both short‐stem and conventional‐stem hips (24.8° ± 7.5° vs. 23.8° ± 6.1°, *p* = 0.313).

**Table 1:** Baseline characteristics of the donor population and postoperative outcomes (*n* = 12)

Variable	Short stems	Conventional stems	P value*
Age (years), mean ± *SD*	86.2 ± 11.7		n/a
Gender, male/female	6/6		n/a
BMI (kg/m^2^), mean ± *SD*	23.1 ± 3.0		n/a
Preoperative antetorsion angle (°), mean ± *SD*	24.8 ± 7.5	23.8 ± 6.1	0.313
Postoperative antetorsion angle (°), mean ± *SD*	23.0 ± 5.5	13.5 ± 7.1	<0.001

Abbreviation: BMI, body mass index, *SD*, standard deviation.

* *p* values determined using paired *t* test.

**Figure 2:**
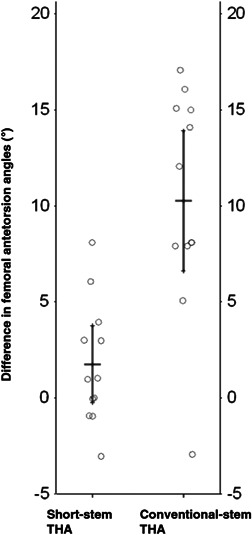
Mean pre‐ and post‐operative differences in femoral antetorsion angle (°) with 95% CI in short‐stem and conventional‐stem THA. THA, total hip arthroplasty

The mean postoperative femoral antetorsion angle was 23.0° ± 5.5° in short‐stem and 13.5° ± 7.1° in conventional‐stem hips (Table 1). Short‐stem hips had a small but nonsignificant difference in femoral antetorsion angles pre‐ and post‐operatively (1.8° ± 3.2°, *p* = 0.109). In sharp contrast, the difference between pre‐ and post‐operative femoral antetorsion angles in conventional‐stem hips was much larger and highly significant (10.3° ± 5.8°, *p* < 0.001) (Figure 2).

## 4 DISCUSSION

In this paired in vitro study, we examined whether calcar‐guided short‐stem THA altered postoperative femoral antetorsion in the same way as conventional‐stem THA. The results confirmed our hypothesis that the femoral antetorsion angle did not change significantly from preoperative values after calcar‐guided short‐stem THA.

Numerous studies exist about the effects of short‐stem THA on primary stability, clinical scores, implant survival, and accurate anatomical reconstruction of the hip with the restoration of postoperative femoroacetabular offset, CCD angles, and leg length.^1^, ^5^, ^6^, ^7^, ^8^, ^9^, ^10^, ^11^, ^21^, ^22^, ^23^, ^24^ Many of these have shown excellent midterm clinical outcomes in these areas.^1^, ^5^, ^6^, ^7^, ^8^, ^9^, ^10^, ^11^, ^21^, ^22^ However, only limited evidence is available on the effect of short‐stem THA on femoral antetorsion. Addressing this topic, we found that short‐stem THA preserved postoperative femoral antetorsion angles better than conventional‐stem THA. Confirming our results, the authors of a recently published cadaveric study also found better restoration of femoral antetorsion with a metaphyseally anchored short‐stem prosthesis compared with a short straight‐stem and a conventional‐stem prosthesis implanted in femoral specimens.^25^ However, in contrast to Ezechieli et al., our study was performed on full‐body cadavers, which represents a more realistic model taking into account the effect of the soft tissues on the femoral antetorsion during implant bed preparation and insertion. Further in line with our findings, Müller et al. also found that conventional‐stem THA resulted in a significantly lower postoperative femoral antetorsion angle than the preoperative value (7.4° vs. 24.9°, *p* < 0.001).^4^ Although the loss of femoral antetorsion did not affect 1‐year clinical outcomes in their study, long‐term clinical significance of this phenomenon remains unknown.

Due to the curved design in calcar‐guided short stems, an individual stem positioning is possible in a wide range of different varus and valgus alignments alongside the medial calcar.^17^ Moreover, the curved design of the short‐stem prosthesis used in this study allows for better restoration of the physiological femoral offset than conventional stems.^7^ Implant positioning usually spares the femoral neck, with the implant guided along the calcar. This anatomical landmark, with metaphyseal fixation, makes it possible to adapt to particular anatomical situations.^7^

With conventional stems, the surgeon's estimation of the femoral component antetorsion is only approximate, and the surgeon can influence the antetorsion by twisting the stem more anteriorly.^4^ With short stems, however, implantation is guided more by the preserved femoral neck.^7^ In addition, femoral antetorsion is influenced by factors such as the anterior bowing, individual anatomy, and original anatomical antetorsion of the femur making femoral component alignment challenging with conventional stems.^4^ In fact, Müller et al. found that conventional‐stem THA resulted in femoral component alignment within 10° in only 50% of the study population.^4^

Improper component torsion of the hip is known to limit the range of motion and increase the risk of complications, such as impingement, instability, dislocation, and component wear.^13^, ^14^, ^26^ Short femoral stems may reduce the risk of these complications due to their ability to accurately reconstruct the hip joint and restore hip biomechanics. In fact, the Australian National Joint Registry reported a lower cumulative incidence of dislocation with short stems than with conventional stems over a 15‐year period.^27^ We suspect that the lower dislocation rates with short‐stem THA could be attributed to the good restoration of femoral antetorsion we saw in our study. However, a possible correlation between femoral antetorsion and dislocation rates, along with other complications and clinical outcomes, requires clinical investigation.

We acknowledge some limitations of our study. First, we used a limited number of samples. That said, 12 cadavers were found to be sufficient to reach statistical significance, according to our sample size estimation. Second, we used Thiel‐fixed instead of fresh‐frozen cadavers. However, Thiel‐fixed cadavers have a texture similar to fresh‐frozen cadavers and offer several advantages, including slower tissue deterioration and the absence of disease transmission.^28^ Thiel‐fixed cadavers are therefore considered suitable for use in orthopedic applications.^28^ Third, because femoral antetorsion can largely depend on the surgical technique, results may vary across clinics. Finally, we used three‐dimensionally printed short stems and trial heads to minimize imaging artifacts, and therefore, our results may be applicable only to the stems evaluated. However, we did use the same procedures, approach, and instrumentation as in a standard in vivo THA, making the procedure comparable to a real‐life clinical scenario. Although polyphenylsulfone stems are smoother than metallic stems due to their nonporous surface, a good press‐fit can be achieved when using the right size. In this study, the appropriate size of the implants was chosen preoperatively.

Nevertheless, our study has several strengths. In particular, all surgeons involved in this study practice in the same clinic and therefore use the same surgical technique, limiting any technical differences. Additionally, we followed the Waidelich method for femoral antetorsion measurement using CT images in transverse planes. Due to the fixed anatomical landmarks, available both pre‐ and post‐operatively, this method is known to have high accuracy and reproducibility, as well as low intra‐ and inter‐observer variability.^20^

In conclusion, our study demonstrated that calcar‐guided short‐stem THA effectively restored femoral antetorsion. However, whether the lower dislocation rates of short‐stem implants result from the good restoration of femoral antetorsion warrants further exploration.

## AUTHOR CONTRIBUTIONS

All authors contributed equally to data collection, analysis, and write‐up. All authors have read and approved the final submitted manuscript.

## CONFLICT OF INTERESTS

The authors declare that there are no conflict of interests.
